# Prolonged Low Flow Reduces Reactive Hyperemia and Augments Low Flow Mediated Constriction in the Brachial Artery Independent of the Menstrual Cycle

**DOI:** 10.1371/journal.pone.0055385

**Published:** 2013-02-05

**Authors:** Mark Rakobowchuk, Emily R. Parsloe, Sarah E. Gibbins, Emma Harris, Karen M. Birch

**Affiliations:** 1 Department of Biological Sciences, University of Essex, Colchester, United Kingdom; 2 Medicine and Veterinary Sciences, University of Cambridge, Cambridge, United Kingdom; 3 Multidisciplinary Cardiovascular Research Centre, University of Leeds, Leeds, United Kingdom; The University of Manchester, United Kingdom

## Abstract

Non-invasive forearm ischemia-reperfusion injury and low flow induced vascular dysfunction models provide methods to evaluate vascular function. The role of oestrogen, an endogenous anti-oxidant on recovery from ischemia-reperfusion injury has not been evaluated nor has the impact of prolonged low flow on vascular function been established. Eight healthy women (33±10 yr) attended the lab during the follicular, ovulatory and mid-luteal phases of their menstrual cycles. After 30 minutes of rest, brachial artery vascular function was assessed by ultrasound measurements of diameter changes during 5 minutes of forearm ischemia and 3 minutes after. Subsequently, a 20-minute forearm ischemia period was completed. Further, vascular function assessments were completed 15, 30 and 45 minutes into recovery. Flow-mediated dilation, low-flow-mediated constriction, and reactive hyperaemia proximal to the area of ischemia were determined. Flow-mediated dilation was reduced at 15 minutes of recovery but recovered at 30 and 45 minutes (PRE: 7.1±1.0%, POST15∶4.5±0.6%, POST30∶5. 5±0.7% POST45∶5.9±0.4%, *p*<0.01). Conversely, low-flow mediated constriction increased (PRE: −1.3±0.4%, POST15: −3.3±0.6%, POST30: −2.5±0.5% POST45: −1.5±0.12%, *p*<0.01). Reactive hyperaemia was reduced throughout recovery (*p*<0.05). Data were unaffected by menstrual phase. Prolonged low flow altered vascular function and may relate as much to increased vasoconstriction as with decreased vasodilation. Reductions in anterograde shear and greater retrograde shear likely modulate the brachial artery response, but the reduced total shear also plays an important role. The data suggest substantial alterations in vascular function proximal to areas of ischemia with potential clinical implications following reperfusion.

## Introduction

The forearm ischemia-reperfusion (I/R) model has been used to create an induced arterial vascular endothelial injury in several recent studies and has provided insightful information about potential interventions to attenuate the associated dysfunction or prevent the injury [1]–[5]. Specifically, the associated dysfunction may be attenuated by intra-arterial vitamin C infusion [1] whereas remote or distant prior ischemia [2], [4] and sildenafil [6] are thought to attenuate the magnitude of the injury. Each of these treatments impacts nitric oxide bioavailability through the scavenging of reactive oxygen species [1], activation of endogenous antioxidant systems [2]–[4], or through the inhibition of cGMP degradation by cGMP-specific phosphodiesterase type 5. The later limits opening of mitochondrial K_ATP_ channels that then lead to apoptosis [5], [6].

Blood flow dynamics and shear rates are altered upstream of a site of ischemia. In models where endothelial cells are located proximal to an area of ischemia, altered gene expression of these endothelial cells is apparent and includes reduced expression of eNOS (endothelial nitric oxide synthase), extracellular and copper-zinc superoxide dismutases, and prostaglandin I_2_ synthase, and increased expression of endothelin-1 and procoagulant factors [7]. However, in vivo prolonged low flow in humans has rarely been studied and the time course of altered vascular function following low flow interventions is unknown.

In studies addressing prolonged low flow using venous congestion, distal cuff inflation, or I/R induced endothelial alterations, repeated flow-mediated dilation (FMD) protocols are used to measure vascular endothelial function [2], [3], [6], [8]. The procedure involves forearm occlusion for a 5-minute period and subsequent measurement of the dilation of the artery under observation. These manipulations involve an initial measurement of FMD prior to the intervention (at least 10 minutes prior), a 20-minute upper or lower arm occlusion period, and subsequent FMD protocols throughout recovery. As described, many interventions improve recovery from these manipulations [2]–[6], [9]. Specifically, exercise training in sedentary middle–aged participants attenuated endothelial dysfunction induced by distal occlusion [8]. Devan et al. (2011) suggest this relates to enhanced NO bioavailability, reduced oxidative stress and inflammation.

In females, endogenous 17-β estradiol may also confer protection against prolonged low flow (PLF) or I/R injury via enhanced NO bioavailability and scavenging of reactive oxygen species [10]. Fluctuations of 17-β estradiol and progesterone throughout the normal menstrual cycle may impact the magnitude of PLF or I/R injury and the kinetics of recovery. As well, since reperfusion causes significant tissue damage through calcium loading, inflammation and a surge of reactive oxygen species production, it may be that prior exposure to 17-β estradiol would limit these effects and offer some degree of protection. To address the hypothesis that endogenous fluctuating hormonal status may impact recovery of vascular function after prolonged low flow we evaluated this parameter prior to and following this intervention at 3 points during the normal menstrual cycle.

A second purpose of this study was to comprehensively evaluate brachial artery vascular function in response to a distal low flow stimulus. Current guidelines for FMD emphasize measuring both arterial shear rates and diameters throughout the protocol [11], [12] however; few studies have followed these recommendations when evaluating I/R or PLF [2], [3], [6], [8] with two studies reporting only the peak to baseline flow ratio as the stimulus for dilation as unchanged [2], [3]. In addition, complementary information may also be gathered during comprehensive vascular evaluations including the magnitude of the vasoconstriction of the brachial artery during distal arterial occlusion. Specifically, this low-flow mediated constriction (L-FMC) provides an index of basal endothelial contribution to vasomotor function [13], which may be partially mediated by endothelin-1 [14] or through inhibiting endothelial derived hyperpolaring factor (EDHF) and cyclooxygenase products (e.g. prostaglandins) [15]. By measuring L-FMC and total vascular reactivity, the absolute sum of L-FMC and FMD, we can determine whether there is truly a reduction in endothelial vasomotor function or in fact there is an altered endothelial contribution to basal vasomotor function following PLF.

## Methods

### Ethics Statement

The experimental procedures and potential risks were explained prior to the study when participants attended an initial familiarization session. All participants provided written informed consent to participate. The Faculty of Biological Sciences Ethics Board at the University of Leeds approved the experimental protocol, which conformed to the Declaration of Helsinki.

### Participants

Healthy women (n = 8) volunteered for the study (Mean ± SD, age: 33±10, SBP: 115±11, DBP: 73±9, HR: 64±8). Participants were: (a) free from apparent cardiovascular, pulmonary or metabolic disease and not taking medication. Other exclusion criteria included pregnancy and smoking as assessed through initial screening. All had self-reported menstrual cycles lasting between 28 and 32 days and had regular menstrual cycles without the use of hormonal contraceptive preparations for the previous 6 months.

### Study Design and Procedures

Participants were initially asked to provide an estimate of their regular menstrual cycle length and only those who had reliable (within 2–3 days) menstrual cycles were included in the study. Participants reported the first day of their cycle (the onset of menstrual bleeding) and subsequent testing procedures were determined from this date. Specifically, early follicular (day 2–4), ovulation (Day 12–14) and mid-late luteal phase (Day 21–28) were chosen to cover three instances when oestrogen and progesterone levels would be altered. As such, day 1 represented the first day of menstrual blood loss, the follicular phase where both hormone concentrations were low, ovulation where estrogen concentration was high and progesterone low, and mid-late luteal where both hormone concentrations were high. Participants attended the temperature controlled (22–24°C) laboratory on these three occasions in sequential order; however, the experimenter was blinded to this via random initiation of the first visit. Thus, the experimenters were blind to menstrual cycle phase. Participants were also instructed to abstain from strenuous exercise for at least 24 hours, and arrived at the laboratory in the morning following an overnight fast and abstinence from caffeine. This was verbally confirmed at each session.

**Figure 1:**
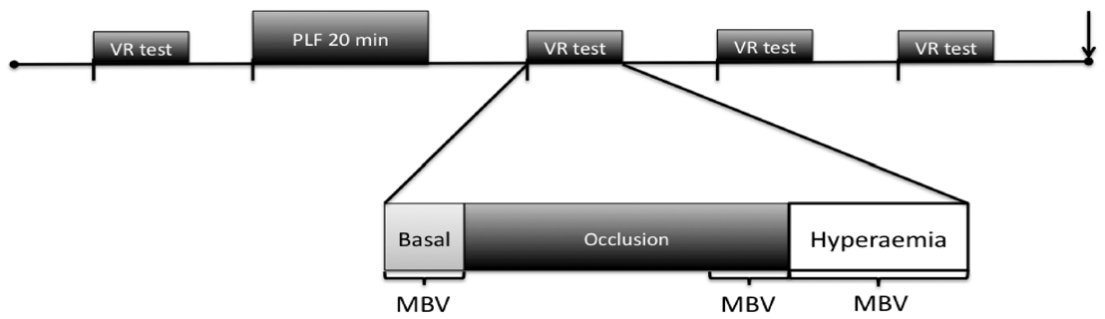
Schematic representation of a testing session timeline. Participants arrived at the laboratory and rested supine for 20 minutes prior to a brachial artery vascular function assessment (VF test). A ten-minute recovery period was immediately followed by prolonged low flow (PLF 20 min) and subsequent VF tests were completed at 15, 30 and 45 minutes into recovery. A venous blood sample was obtained at 120minutes (↓). The VF test mean blood velocity (MBV) measurements are highlighted in the expansion. Basal MBV was measured prior to cuff inflation; occlusive MBV 30s prior to cuff deflation; and hyperaemic MBV was measured continuously for 90s after cuff release as illustrated.

Figure 1 illustrates the time-line of each experimental session. Briefly, participants were initially instrumented in the supine position after which a 20-minute resting period was completed. Following this an assessment of arterial vascular function was conducted on the right brachial artery using the FMD protocol (i.e. PRE). The arm was maintained in this abducted position throughout each experimental session. Following 10 minutes of recovery from the first assessment of vascular function, a 20-minute forearm occlusion and thus prolonged upstream low flow was induced via the same cuff as previously outlined by DeVan et al. (2011), which was maintained at 250–300 mmHg. Further vascular function testing was repeated at 15, 30 and 45 minutes of recovery (i.e. POST15, POST30, POST45). An identical timeline was replicated at the subsequent 2 visits.

### Vascular Endothelial Function Testing Procedures

Comprehensive assessment of brachial vascular function was conducted according to previous work in this laboratory using semi-automated edge detection for diameter analysis and fast Fourier transform of raw audio data to determine mean blood velocity [16]. Briefly, a blood pressure cuff was placed around the forearm aligned with the medial epicondyle at all testing sessions and at all time points during the experimental sessions. Arterial shear rates and blood flow were monitored using a 7 Mhz linear array Doppler ultrasound in duplex mode (Aspen, Acuson, Siemens Medical, Camberley, UK). Arterial diameters were acquired for 20 heart cycles in the basal state prior to each 5 minute occlusion period and for 210 heart cycles from 30s prior to, until ∼180s post cuff deflation. All participants had resting heart rates of below 70 bpm and all participants reached maximal dilation within the first 90s after cuff deflation. Arterial diameters were purposefully acquired prior to cuff deflation so that L-FMC could be determined alongside traditional FMD parameters and total vascular reactivity (TVR) calculated as the absolute sum of L-FMC and FMD [16]. This procedure was repeated at 15, 30 and 45 min after the PLF. Heart rate and brachial arterial blood pressures were also acquired at each time point.

Mean blood velocity (MBV) was determined for 30s in the basal condition before the 5 min forearm occlusion, for 30s prior to cuff deflation (i.e. during occlusion), and throughout reactive hyperaemia for 60s following cuff deflation. Shear rates were calculated (8×MBV/arterial diameter at the measurement time) and the area under the shear rate curve was determined for 60s (AUC_shear60s_), while the peak blood flow after cuff deflation was also determined (peak MBV×πr^2^, where r is the diameter immediately after cuff deflation). Finally, retrograde and anterograde area under the MBV curves for 30s in both basal and occlusive conditions at each time point within a session were calculated. These waere used to determine changes in arterial shear patterns from basal conditions prior to cuff inflation, compared to conditions of distal arterial occlusion.

After completion of the protocol, an antecubital venous blood sample was collected, centrifuged, and the plasma was stored in a freezer at −80°C until analysis. Determination of serum 17-β estradiol (Estradiol EIA kit, Cayman Chemical Item number 582251, Cambridge, UK) involved standard ELISA while progesterone was determined by competitive immunoassay and measured directly by chemilminescent methods using an ADVIA Centaur Progesterone Assay (Bayer). Measurements were performed in the Pathology Research and Development lab of Leeds Medical Trust, Leeds, West Yorkshire.

### Statistical Analyses

Data were assessed for normal distribution using Kolmogorov-Smirnov test. Absolute and relative FMD, absolute and relative L-FMC, TVR, peak blood flow, 60s shear rate AUC, basal and occlusive shear, and basal and occlusive anterograde and retrograde 30s MBV AUC were analyzed by 2 way-repeated measures ANOVA with “menstrual phase” and “time” treated as within subject variables. When a significant effect was noted Bonferroni corrected t-tests were used to determine specific differences. Significance for all analyses was accepted as *p*≤0.05. All values are presented as mean ± standard deviation. Analyses were performed using statistical software (SPSS Version 18.0, IBM Corporation, Somers, NY, USA). The day-to-day repeatability (CV) for FMD, L-FMC, and TVR is 15%, 20%, and 12% (n = 8), respectively. The absolute day-to-day difference is 1.1%, 1.4%, and 1.7%, respectively in this laboratory by MR who conducted all the testing sessions. Power calculation based on previous work in this laboratory suggested a sample size of 6 participants would be required to see a 1% change in FMD with a standard deviation of the sample of 2% (effect size of 0.71) with a probability of 0.05 and a beta of 0.80. Thus adequate statistical power was aided by using a sample of 8 in the present study.

## Results

**Figure 2:**
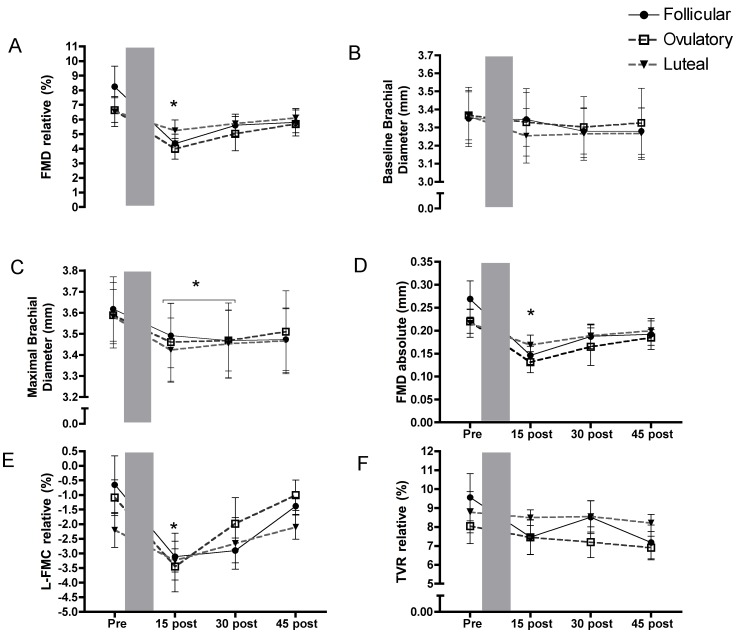
Vascular function alterations. a) Relative flow mediated dilation (FMD) was reduced after low flow induced vascular dysfunction (PLF) (shaded area) and remained suppressed throughout recovery although it was not different from PRE at 30 and 45 minutes. b) Diameter prior to each vascular function test was unchanged compared with PRE while c) maximal diameter was blunted and as a result d) absolute FMD was reduced at 15 minutes after PLF. e) Conversely L-FMC was augmented at 15 minutes after PLF but recovered by 30 minutes. f) As a result of augmented L-FMC and reduced FMD, TVR was unaltered throughout the protocol. No significant differences were noted within the menstrual cycle. * Indicates a significant main effect for time (*p*≤0.05) from specifically difference compared to PRE PLF (post hoc comparison). FMD is flow-mediated dilation, L-FMC is low flow mediated constriction, and TVR is total vascular reactivity.

Participants were studied at 3 distinct points in the menstrual cycle (17**-**β estradiol: Follicular: 147±42 pg/ml Ovulation: 217±32 pg/ml Luteal: 165±35 pg/ml, progesterone: Follicular: 2.3±0.8 nmol/L Ovulation: 4.0±2.1 nmol/L Luteal: 16.8±4.3 nmol/L). A mean reduction in relative FMD (36%, *p*<0.05) at 15 minutes post PLF was observed in these women that was not influenced by menstrual cycle phase (Fig. 2a). Values POST30 and POST45 after PLF did not significantly differ from PRE. The pattern of recovery was not influenced by menstrual phase (p>0.05). Basal diameters at each time-point were also similar (Fig. 2b) and as a result, peak diameters and absolute FMD were reduced following PLF at POST15 (Fig. 2c,d). Finally, time to peak dilation showed a similar pattern with a reduction at POST15 and a return to values not different from PRE at POST30 and POST45. There was no impact of the menstrual cycle phase on this value (p>0.05).

L-FMC displayed a similar pattern following PLF to FMD alterations with a more pronounced constriction at POST15, 149% of PRE values (*p*<0.05) that was not influenced by menstrual cycle phase (Fig. 2e). The absolute L-FMC change from PRE to POST15 was 0.06 mm, while the absolute change in FMD was 0.09 mm. As a result, there were no alterations in TVR (Fig. 2f) after PLF although a trend for reduced TVR was apparent at POST45 but this did not reach statistical significance (p = 0.10).

### Shear Rates upon Cuff Deflation

**Figure 3:**
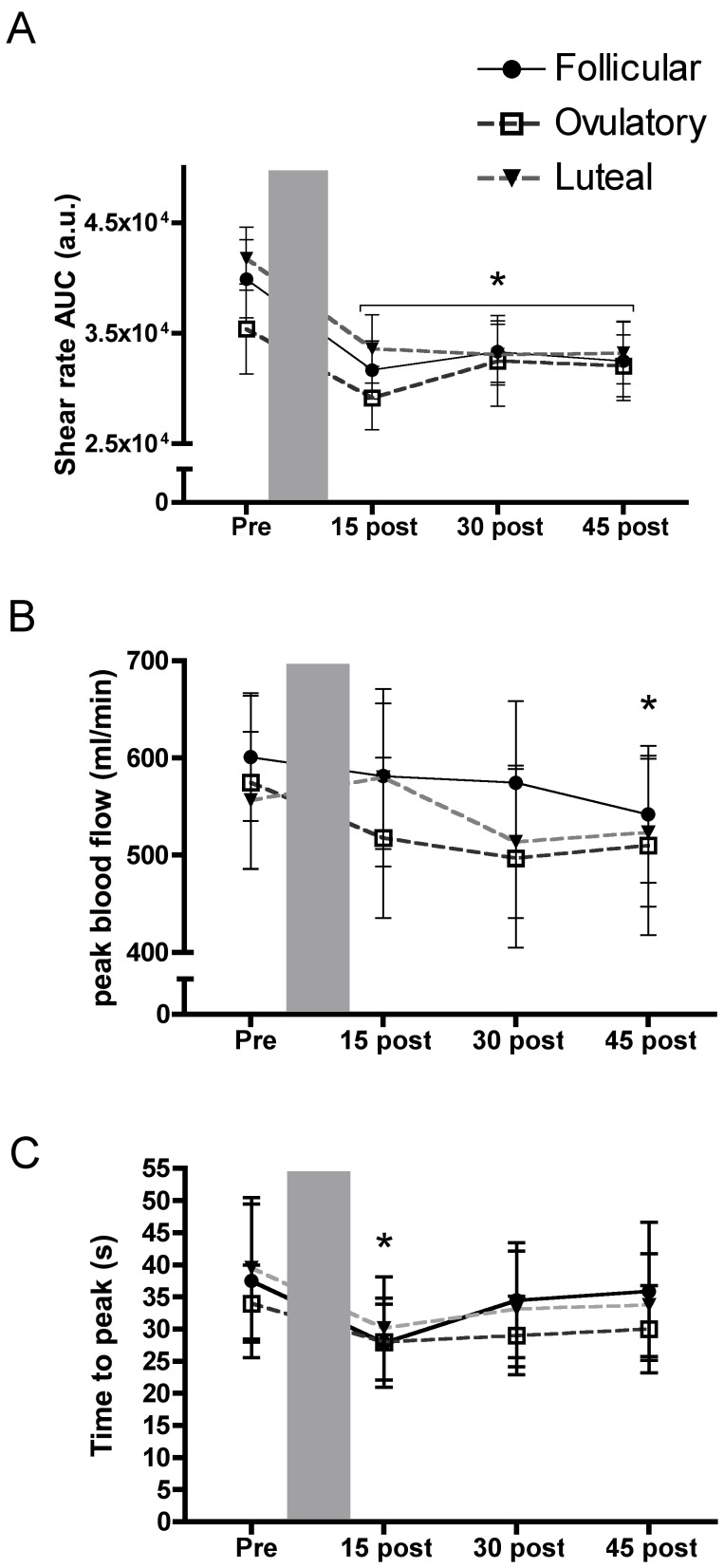
Shear rate responses after cuff deflation. a) 60s brachial artery reactive hypaeremic shear rate area under the curve (AUC) were reduced throughout the recovery period compared with measures taken prior to the prolonged low flow (PLF) (shaded area). b) Peak reactive hyperaemic blood flow was unaltered by PLF whereas c) time to peak dilation was reduced at 15 minutes of recovery but recovered by 30 minutes. There were no significant differences within the menstrual cycle. * Indicates a significant difference (*p*≤0.05) from PRE PLF (main effect for Time) determined by post hoc analysis. AUC is the area under the curve.

The area under the shear rate curve (AUC_shear60s_), determined over the first 60s after cuff deflation during each vascular function measurement (i.e. PRE, POST15, POST30 and POST45), was unaffected by menstrual cycle phase; however at all time points after PLF, there was a reduction of between 15–22% (*p*<0.05) compared with PRE. This would suggest a reduced shear stimulus (see Fig. 3a,b) and possibly endothelial dysfunction at the level of the resistance vessels following PLF. Peak blood flow upon cuff release (peak reactive hyperaemia) was reduced at POST45 compared to PRE, however menstrual cycle phase had no impact (Fig. 3c).

### Basal and Occlusive Mean Blood Velocity

**Figure 4:**
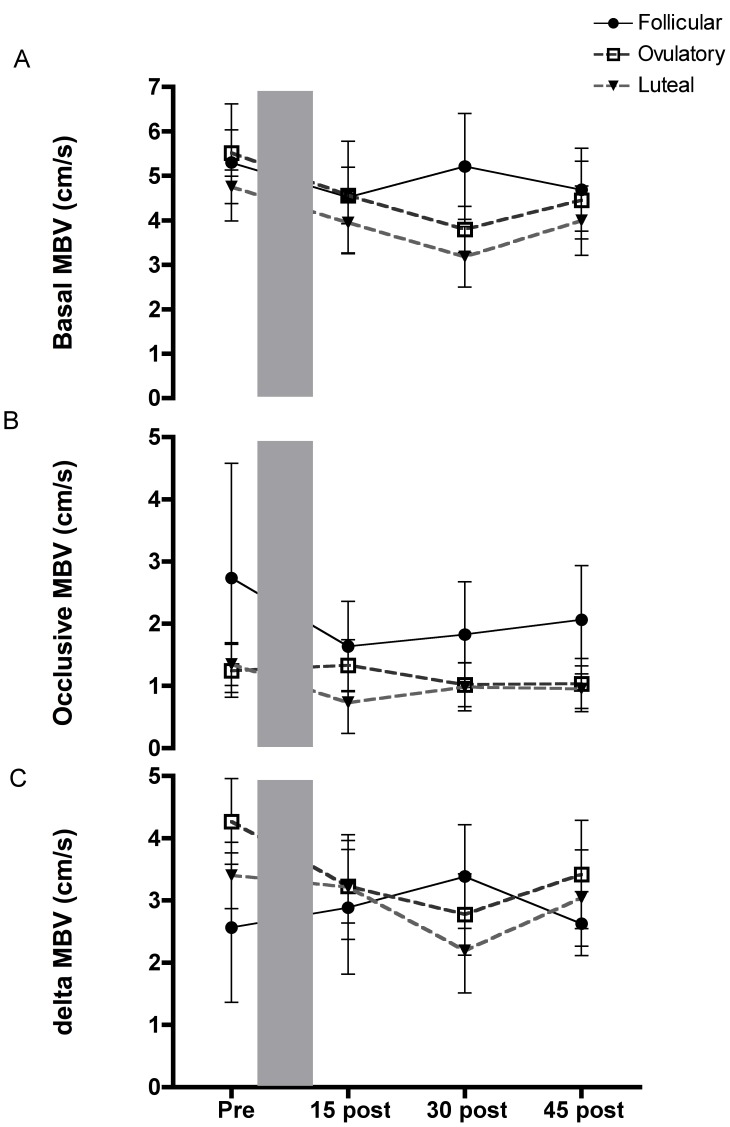
Basal and distal occlusive brachial artery shear. a–b) basal brachial mean blood velocity and brachial mean blood velocity measured when distal occlusion was applied was not altered by prolonged low flow (shaded area). c) Change in mean blood velocity upon cuff inflation was unaltered. MBV is the mean blood velocity.

Mean blood velocities monitored prior to each cuff inflation (basal) and 30s prior to cuff deflation (occlusive) during the vascular function tests (i.e. FMD and L-FMC assessments) were not different when they were compared between time points (i.e. PRE vs. Post 15 etc; Fig. 4a,b). Mean blood velocities were reduced when the forearm cuff was inflated during each vascular function test, but the magnitude of this reduction was similar at all time points (Fig. 4c, *p*>0.05). Similar to all other parameters there was no effect of menstrual cycle phase.

### Anterograde and Retrograde Shear Induced by Occlusion

**Figure 5:**
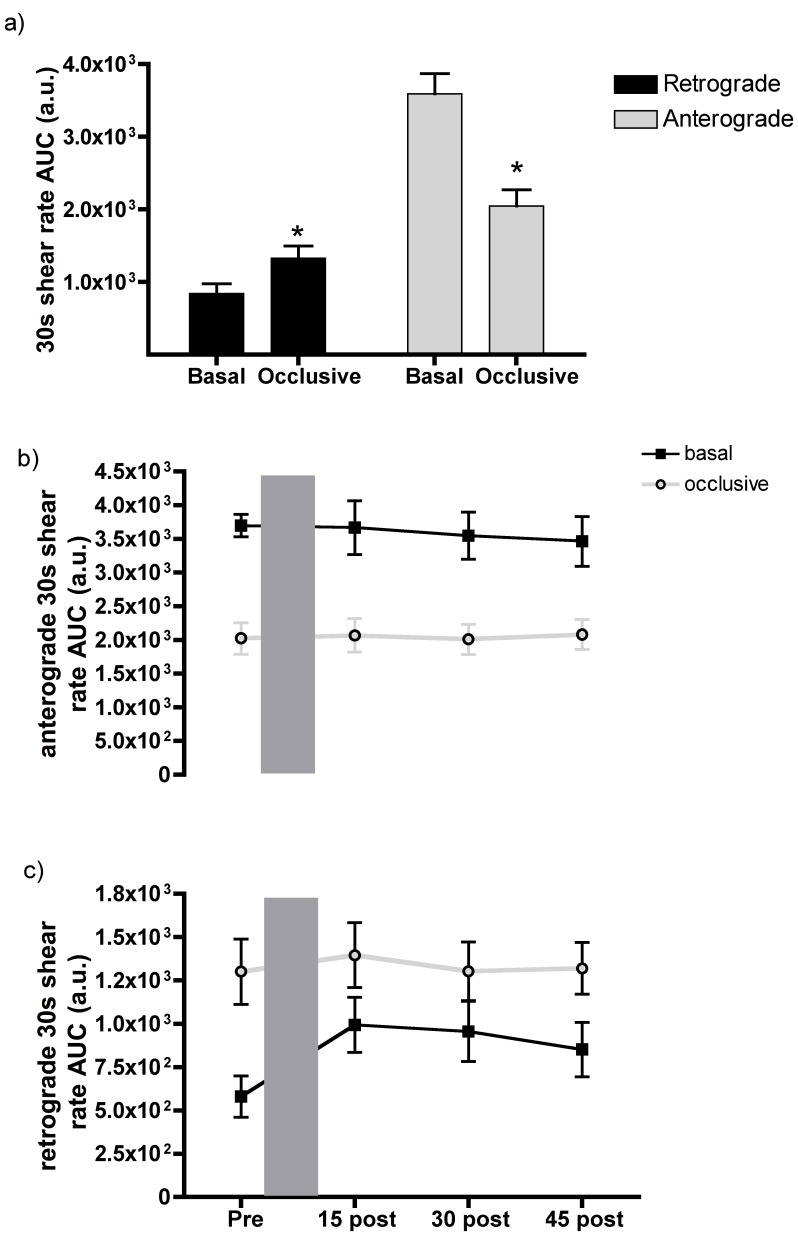
Basal and distal occlusive brachial anterograde and retrograde shear rates. a) An increase in retrograde and a concomitant decrease in anterograde shear rate were evident upon distal arterial occlusion. This graph illustrates the mean of all vascular function responses. b) Basal and occlusive anterograde AUC shear was not altered by the prolonged low flow (PLF) (PRE vs. POST15, 30 or 45). Similarly c) basal and occlusive retrograde AUC shear were not altered by the PLF. Since menstrual cycle phase had no impact, mean responses are illustrated in graphs a and b. * Indicates a significant difference (*p*≤0.05) between basal and occlusive velocities. AUC is the area under the curve.

The area under the anterograde and retrograde shear rate curves were determined over a 30s period prior to each cuff inflation (basal) and deflation (occlusive) during the vascular function assessments (FMD and L-FMC measures). There was a significant increase in retrograde AUC shear rate in the basal state (*p* = 0.02, Fig. 5c) suggesting more retrograde flow after PLF compared to PRE. However, post hoc analyses revealed only a tendency for higher retrograde flows at POST15 compared to PRE (*p* = 0.06). Anterograde AUC shear rate in the basal condition before the vascular function assessment was unaltered by PLF (*p*>0.05).

During each 5 min cuff occlusion, there was a consistent increase in retrograde AUC shear rate (*p*<0.05) compared to basal levels (Fig. 5c). Concurrently, anterograde AUC shear rate was significantly reduced at each vascular assessment when there was distal occlusion and this was not altered by PLF (Fig. 5b).

## Discussion

The most important findings of this study were that menstrual cycle phase had no impact on the magnitude or time course of vascular recovery following a period of prolonged arterial low flow induced proximal arterial injury and distal microvascular I/R injury. We have also shown that this manipulation cannot simply be described by alterations in FMD, since the observed reduction in FMD coincided with a concomitant increase in L-FMC. We observed a pronounced reduction in arterial area under the shear rate curve induced by 5 minutes of arterial occlusion after PLF that was not related to the peak of the hyperaemic response, but was likely a function of the kinetics of this response (i.e. the AUC for the hyperaemia). Finally, TVR was not altered following PLF suggesting an alteration in the vasodilation to vasoconstriction relationship that was not influenced by oestrogen availability.

There are several important implications of this study. First, naturally occurring menstrual cycle alterations in an endogenous eNOS activator, namely 17-β estradiol, has no apparent impact at physiological levels on recovery from PLF, and suggests the downstream effects of oestrogen related alterations in NO are negated by low flow and the subsequent oxidative stress induced by reactive hyperaemia. Second, L-FMC may provide additional information about altered vascular function and may indicate an alteration in basal endothelial contribution to vasomotor function before and after PLF [17]. For instance enhanced L-FMC may indicate that any alterations in FMD might not be due to reduced vascular function, but greater vessel relaxation after the PLF thus blunting apparent FMD responses. Alternatively, enhanced L-FMC may be an additional manifestation of endothelial dysfunction as suggested by Weissgerber et al. [18].

### Low Flow Induced Vascular Function Changes

Previous work has established that the I/R model alters pharmacologically induced resistance vessel endothelial dependent vascular function [1], [5], [9], [19] and that endothelial independent vascular function is unaltered [3]. In addition, the endothelial dysfunction induced by I/R is attenuated by ischemic preconditioning either in the limb exposed to I/R [4] or at another site [3], ischemic post-conditioning [2], and interventions that alter K_ATP_ channel function [5]. Acute intra-arterial antioxidant administration also attenuates this injury [1]. Similar mechanisms may be involved in endothelial dysfunction induced by PLF.

The reductions in FMD after PLF in the present study may result from mechanisms related to NO bioavailability in the brachial arterial segment. Alternatively, the reduced FMD could be a function of the observed reduction in shear after cuff deflation if one ascribes to the theory of shear dependent dilatory responses in the forearm [20], although this has been challenged [21]. The attenuated reactive hyperaemia in the current study, both measured as the area under the shear rate curve and peak blood flow, likely indicate microvascular endothelial dysfunction explained by a mechanism related to the opening of the mitochondrial permeability transition pore resulting in inadequate ATP production and subsequent apoptotic signaling by cytochrome c in endothelial cells [3], [5]. Also, augmented reactive oxygen species production and reduced NO bioavailability, and/or resistance vessel obstruction by activated neutrophils [4] and/or a more thrombogenic environment [19] could contribute to this reduced shear stimulus.

### Alteration of Flow Pattern May Alter FMD and L-FMC Responses

Measurement of L-FMC is a unique aspect of the current study since this measure provides additional insight about the endothelial contribution to vasomotor function in basal conditions. As well, changes in L-FMC are suggested to be mediated through mechanisms independent of NO such as endothelin-1 [14], cyclooxygenase products, and endothelial hyperpolarizing factor (EDHF) [15] providing further insight into vascular function. Our results suggest that one or more of these pathways were altered after PLF and reperfusion, potentially due to the prolonged increase in retrograde flow induced by distal arterial occlusion. It is also possible, that retrograde flow induced NADPH oxidase activation and greater reactive oxygen species production, which would directly impact vasoconstriction. NADPH oxidase has been suggested as the site of reactive oxygen species production with I/R and a similar mechanism may be involved in the PLF model [22]. Thijssen *et al*. (2009), using varying levels of venous congestion, also elegantly demonstrated how altering retrograde flow reduced FMD responses but they did not evaluate L-FMC and our data demonstrate for the first time that vasoconstrictor function is also altered by prolonged low flow.

### Total Vessel Reactivity is Maintained

TVR is another method of evaluating the magnitude of vascular function by assessing the combined L-FMC and FMD as a single value [17]. This measure was unaltered by PLF but was a function, as described, of reduced FMD and augmented L-FMC. Since basal diameter was unchanged, an alteration in basal vessel tone is less likely. The more likely explanation is that the altered vessel milieu caused by oxidative stress and altered endogenous vasoactive substances blunted FMD but conversely augmented L-FMC.

### Relation to the Forearm Ischemic-reperfusion Injury Model

The distinct advantage of the distal cuff placement is two-fold, but mechanistically the result of the PLF may differ from protocols involving upper arm occlusion. One advantage of the cuff placement distal to the site of measurement is that it enables continuous visualization of the vessel being investigated. In the present study, the probe remained in place throughout the 2-hour protocol, which ensured precise interrogation of the same portion of the artery and enhanced reproducibility of measures. Second, we were able to determine not only FMD responses but also L-FMC and TVR, which would otherwise be impossible with upper arm occlusion.

Our data suggest that measurements made proximal to the occlusion site reflect a reduced arterial shear stimulus upon cuff deflation, altered shear profiles and greater oscillatory shear that contribute to reduced endothelial dependent dilation and enhanced constriction in the conduit artery. As well, these data suggest that the vessels proximal to the occlusion are also detrimentally impacted and may require further study.

### Limitations

As outlined, this study involved a cohort of female participants, which detracts from the extrapolation of the findings to male cohorts. Blockade studies to determine the underlying mechanism responsible for the alterations in L-FMC would be appropriate. The sample size may limit extrapolation of the findings, however adequate power was maintained as our results illustrate large effect sizes congruent with our a priori sample size calculations. Finally, the impact of repeated vascular function tests has not been established regarding the measurement of L-FMC, however logically if there was an impact of this simple manipulation alone, there would not have been a return to baseline values during recovery from the PLF. As such, we are confident that the greater L-FMC after PLF was a result of the manipulation and not the repeated vascular function tests. We did not assess endothelial independent dilation to confirm previous findings and extend them to the female cohort in this study. Further work to confirm that the menstrual cycle does not impact the response of endothelial independent dilation before and after a PLF stimulus is required.

### Perspectives

This study suggests that PLF augments vasoconstrictor function of arteries proximal to a site of arterial occlusion. Blunted endothelial dependent vessel dilation after PLF may be a function of not only reduced arterial shear induced by reactive hyperaemia, but also by the hemodynamic alterations upstream of the occlusion. Specifically, reduced arterial anterograde shear and increased retrograde shear may be responsible for the endothelial dysfunction. Combined, the results suggest that this PLF model is complex and warrants further investigation into the mechanisms underlying this reaction of the conduit and microvascular circulation.
